# Lifestyle or Environmental Influences and Their Interaction With Genetic Susceptibility on the Risk of LADA

**DOI:** 10.3389/fendo.2022.917850

**Published:** 2022-06-29

**Authors:** Sofia Carlsson

**Affiliations:** Institute of Environmental Medicine, Karolinska Institutet, Stockholm, Sweden

**Keywords:** lifestyle, gene*environment, obesity, smoking, HLA, latent autoimmune diabetes

## Abstract

**Background:**

LADA is a common form of diabetes described as a mix between type 1 and type 2 diabetes. Understanding of how genes and environmental factors interact in the development of LADA is central for future efforts to prevent the disease. This review aims to synthesize the literature on lifestyle factors linked to LADA risk and discuss their potential interaction with genetic susceptibility.

**Findings:**

Current knowledge on environmental risk factors for LADA is primarily based on observational data from Scandinavian populations. Increasing evidence suggest that lifestyle factors promoting type 2 diabetes such as obesity, sedentariness, low birth weight and smoking, is implicated in the risk of LADA. Data from mendelian randomization studies support that the link between LADA and obesity, low birth weight and smoking is causal. Limited evidence indicates that dietary factors including consumption of red meat, coffee and sweetened beverages may increase the risk while consumption of alcohol and omega-3 fatty acids may reduce the risk. Several lifestyle factors, including smoking and obesity, seem to interact with human leukocyte antigen genes associated with autoimmunity, conferring much stronger effects on disease risk among those exposed to both factors.

**Summary:**

Available studies suggest that lifestyle modification has the potential for prevention of LADA, particularly for individuals with high risk of disease such as those with genetic susceptibility. Research into risk factors of LADA is however limited, confirmations are warranted, many factors remain to be explored, and there is a need for intervention studies to assess causality

## Introduction

Latent autoimmune diabetes in adults (LADA) is an intermediate form of diabetes sharing characteristics with both type 1 and type 2 diabetes (1–3). It is characterized by adult onset, autoantibodies, and preserved beta-cell function at the time of diagnosis. LADA was first described in 1977 when it was discovered that a proportion of patients diagnosed with type 2 diabetes had autoimmune markers typically found in type 1 diabetes (4). In 1993, the term “LADA” was suggested by Finnish researchers to describe this subgroup of patients, with an autoimmune form of diabetes that progresses slower to insulin deficiency than type 1 diabetes (5). Since then, the genetic (6, 7) and clinical characteristics (2, 8) of LADA have been described in various populations, confirming its hybrid nature with a genetic and immunological profile resembling that of type 1 diabetes, and a phenotype closely linked to type 2 diabetes. It has been observed that LADA is present in 3-12% of individuals diagnosed with type 2 diabetes, making it the most common form of autoimmune diabetes with adult onset (9). Like type 1 diabetes, LADA seems to be more frequent in Europe than in other parts of the world, including Asia, Africa, and North America (9). It is generally believed that environmental factors, acting on genetic susceptibility, are implicated in the promotion of both type 1 and type 2 diabetes, and much effort has gone into exploring such factors (10, 11). Investigations into the role of environmental factors and their interaction with genetic susceptibility in LADA are much more limited but have slowly accumulated over the last decade (most recently reviewed in 2019 (12)). The aim of this report is to provide an updated synthesis of studies addressing the association between lifestyle factors and LADA risk that will include results from the first gene*lifestyle interaction studies as well as the first Mendelian randomization studies in LADA.

## Definition

The definition of LADA is usually based on three criteria that were proposed by the Immunology of Diabetes Society (13); 1) presence of autoantibodies, typically GADA, which is a basic criterion that distinguishes LADA from type 2 diabetes; 2) adult onset, often defined as 30 years or older; and 3) lack of insulin treatment during at least 6 months following diagnosis to indicate a slow “latent” progression of autoimmunity and separate LADA from type 1 diabetes. The concept of LADA is debated, and the controversy includes whether it is useful to regard LADA as a separate entity (14), with some arguing that it should essentially be viewed as type 1 diabetes with adult onset (15), and others stating that LADA encompasses a mix of type 1 and type 2 diabetes patients (16). This ambiguity is reflected in current guidelines on the classification of diabetes. As an example, the American Diabetes Association classifies LADA as type 1 diabetes in their 2020 guidelines (17), whereas the latest WHO classification from 2019 (18) categories LADA as a hybrid form of diabetes and proposes that the term “LADA” should be replaced by the term “slowly evolving immune mediated diabetes in adults”. Moreover, LADA does not have a unique ICD-code, which limits the possibilities to study LADA using register-based approaches. Elucidating whether LADA has a distinct etiology and risk factors will hopefully aid in determining how significant it is to distinguish LADA as a separate subtype of diabetes.

## Pathogenesis

LADA has an autoimmune pathogenesis characterized by the presence of circulating autoimmune antibodies. Whereas individuals with type 1 diabetes tend to be positive for several autoantibodies, the majority of people affected by LADA show positivity to only one pancreatic autoantibody, which, in most cases, is autoantibodies to glutamic acid decarboxylase (GADA) (19, 20). These antibodies have been documented for up to 10 years prior to diagnosis, which suggest a long preclinical phase of LADA (20, 21). The autoimmune process leading to beta-cell destruction is milder in LADA than in type 1 diabetes, and the patients progress to insulin deficiency at a slower rate. Consequently, there is typically no imminent need for insulin treatment during the first 6 to 12 months following diagnosis. In addition to islet autoimmunity, LADA patients tend to be insulin resistant, and the degree of insulin resistance seems to depend on the degree autoimmunity, so that it is more pronounced in LADA patients with low levels of GADA (22, 23). Indeed, data from the ESTRID study show an inverse association between levels of GADA and insulin resistance as measured by Homeostasis model assessment (HOMA) at the time of diagnosis (12). This is in line with the hypothesis postulated by Naik et al. (24), stating that the degree of autoimmunity may determine at what point in the autoimmune process, LADA will emerge. Based on what is known about the pathogenesis of LADA, it seems possible that environmental or lifestyle factors may promote LADA either by triggering autoimmunity and/or by inducing insulin resistance. Increased insulin resistance, once autoimmunity is present, may accelerate the progression to manifest diabetes by increasing the demand on the beta cells for a compensatory rise in insulin secretion. The influence of such factors may be contingent of genetic susceptibility, and such interactions may be different for susceptibility to type 1 diabetes and type 2 diabetes.

Studies have shown that the phenotype of LADA can range from being very type 1-like with high levels of GADA, pronounced insulin deficiency and fast progression to insulin dependence, to a more type 2-like state with lower levels of GADA indicating less autoimmune activity, a higher degree of insulin resistance and a higher body mass index (BMI) (19, 25, 26). The influence of potential lifestyle risk factors may differ between subtypes of LADA so that those with a more type 2-like phenotype have an etiology that more closely resembles that of type 2 diabetes.

## Genetic Risk Factors

The similarities between LADA and type 1 diabetes are clear from genetic studies (reviewed in 6); a high risk of LADA is conferred by genes in the human leukocyte antigen (HLA) region, known to account for 40-50% of the familial aggregation of type 1 diabetes (27). Like type 1 diabetes, the strongest genetic risk factors for LADA are variation in the MHC class II genes HLA-DQB1 and HLA-DRB1; and the highest risk is conferred by the HLA haplotypes HLA-DRB1*04-DQB1*0302 (“DR4”) and HLA-DRB1*0301-DQB1*0201 (“DR3”) (6). These findings confirm the autoimmune nature of LADA since the HLA genes encode the major histocompatibility complex (MHC) that regulates the immune system. The associations are generally stronger for type 1 diabetes than for LADA, and the effect sizes are also more pronounced for LADA with high levels of GADA (6). However, the key type 1 diabetes-associated HLA genotypes are also associated with LADA with low GADA levels, defined as GADA in the lowest tertile (28) or below median (29), but not with type 2 diabetes (1, 29). In addition, LADA is linked to type 1 diabetes-associated loci outside of the HLA region, including *PTPN22*, *INS*, and SH2B3 and the associations with *PTPN22* and *I*NS appear stronger for LADA with high GADA and for LADA characterized by multiple autoantibodies, respectively (6).

A genetic overlap between LADA and type 2 diabetes has also been observed; some studies have linked LADA to a risk locus on the transcription factor 7-like 2 (*TCF7L2*) gene, the strongest known genetic risk factor for type 2 diabetes (reviewed in 6). Associations with *TCF7L2* are not consistent across studies (30) and may primarily concern less autoimmune forms of LADA with low GADA levels. Studies using genetic risk scores also find that LADA is linked to genetic susceptibility to type 2 diabetes, in addition to type 1 diabetes-susceptibility (30, 31). A family history of diabetes encompasses genetic as well as shared environmental factors and is a strong predictor of diabetes risk. Individuals with first-degree relatives with diabetes have a 2-4-fold increased risk LADA (31–33). Analyses of the relative importance of family history of type 1 diabetes vs. type 2 diabetes support genetic similarities between LADA and type 1 diabetes. A relative risk of six is observed in individuals with first degree relatives with type 1 diabetes, compared to a relative risk of two in those with type 2 diabetes in the immediate family (34).

## Lifestyle or Environmental Factors

### Literature Search

To provide an updated summary of the literature investigating links between environmental or lifestyle factors and the incidence of LADA, a PubMed search of the term “latent autoimmune diabetes in adults” was performed to identify studies investigating modifiable risk factors for LADA. This search generated 598 hits (Dec 28, 2021), but only a small proportion (n =19) of these studies provide data on the incidence of LADA in relation to lifestyle factors (the full list is given as **Supplementary Material**). The paucity of studies is likely due to a shortage of data; few cohorts have information on the incidence of LADA, which requires antibody testing to separate LADA from type 2 diabetes. Moreover, there is no ICD code for LADA and that precludes using hospital registers to identify cases because, in such registers, LADA will be coded as type 1 diabetes if antibodies are measured or most likely as type 2 diabetes if classification is based only on clinical presentation. The studies on environmental or lifestyle risk factors for LADA identified by this literature search were all based on Scandinavian data, either case-control data from the Swedish ESTRID-study (Epidemiological study of risk factors for LADA and Type 2 diabetes) or prospective cohort data from the Norwegian HUNT-study (TrØndelag Health Investigation)-study. These studies are unique because they include incident cases of LADA and type 2 diabetes, population-based controls together with information on lifestyle prior to diagnosis, demographic factors, family history of diabetes, and genetic and clinical characteristics. This provides opportunities to study a wide range of potential risk factors and their interactions with genetic susceptibility, while accounting for potential confounders. In short, the ESTRID study is nested within the ANDIS register and biobank in the Swedish County of Scania (30); since 2010, it has been recruiting incident cases of LADA and type 2 diabetes from ANDIS, together with incidence density sampled controls, randomly selected from the population of Scania (www.ki.se/imm/estrid). Information on potential exposures is collected by questionnaire and through the extensive Swedish Registers. The HUNT-study is one of the largest population-based studies ever conducted. Between 1984 and 2018 more than 100 000 inhabitants of the TrØndelag County participated in health examinations, provided blood samples, and responded to questionnaires in up to four survey waves (35). The latest publication based on ESTRID includes ~600 incident cases of LADA, ~2000 cases of type 2 diabetes, and ~3000 controls collected from 2010-2019 (36). The HUNT-study includes 245 incident cases of LADA and ~3700 cases of type 2 diabetes (36). The effect measures from these studies will be referred to as relative risks (RR) out of convenience, irrespective of whether they were odds ratios (OR) based on case-control data or hazard ratios (HR) based on cohort data or pooled OR/HRs.

Results based on observational data may be influenced by systematic errors such as imperfect or absent adjustment for confounding factors, misclassification of lifestyle exposures, including recall bias, and reverse causation, e.g., if pre-diagnostic symptoms have led to lifestyle changes prior to diagnosis. The mendelian randomization (MR) design allows for stronger causal inferences than observational data because it can minimize systematic errors, including confounding, differential misclassification of exposure, and reverse causation (37). MR studies use genetic variants (most commonly SNPs) as proxies or instrumental variables (IV) for environmental or lifestyle exposures and assess the association between the IV and the outcome of interest (38). The rationale behind the MR design is that genetic variation is assigned randomly at conception and therefore unrelated to confounding factors, and not influenced by the later onset of disease. MR designs are becoming increasingly popular and a PubMed search of the terms “diabetes” and “mendelian randomization” or “mendelian randomisation” identifies 842 papers published between 2005 and 2021. Nevertheless, none of these studies addressed LADA. To fill this knowledge gap, we have recently conducted the first two-sample MR study in LADA. We used outcome data from a publicly available genome-wide association study (GWAS) on LADA, including >2600 cases of European ancestry (27), together with IVs for birth weight, adult BMI, and smoking identified through separate large consortia-GWAS conducted in European populations.

**Figure 1:**
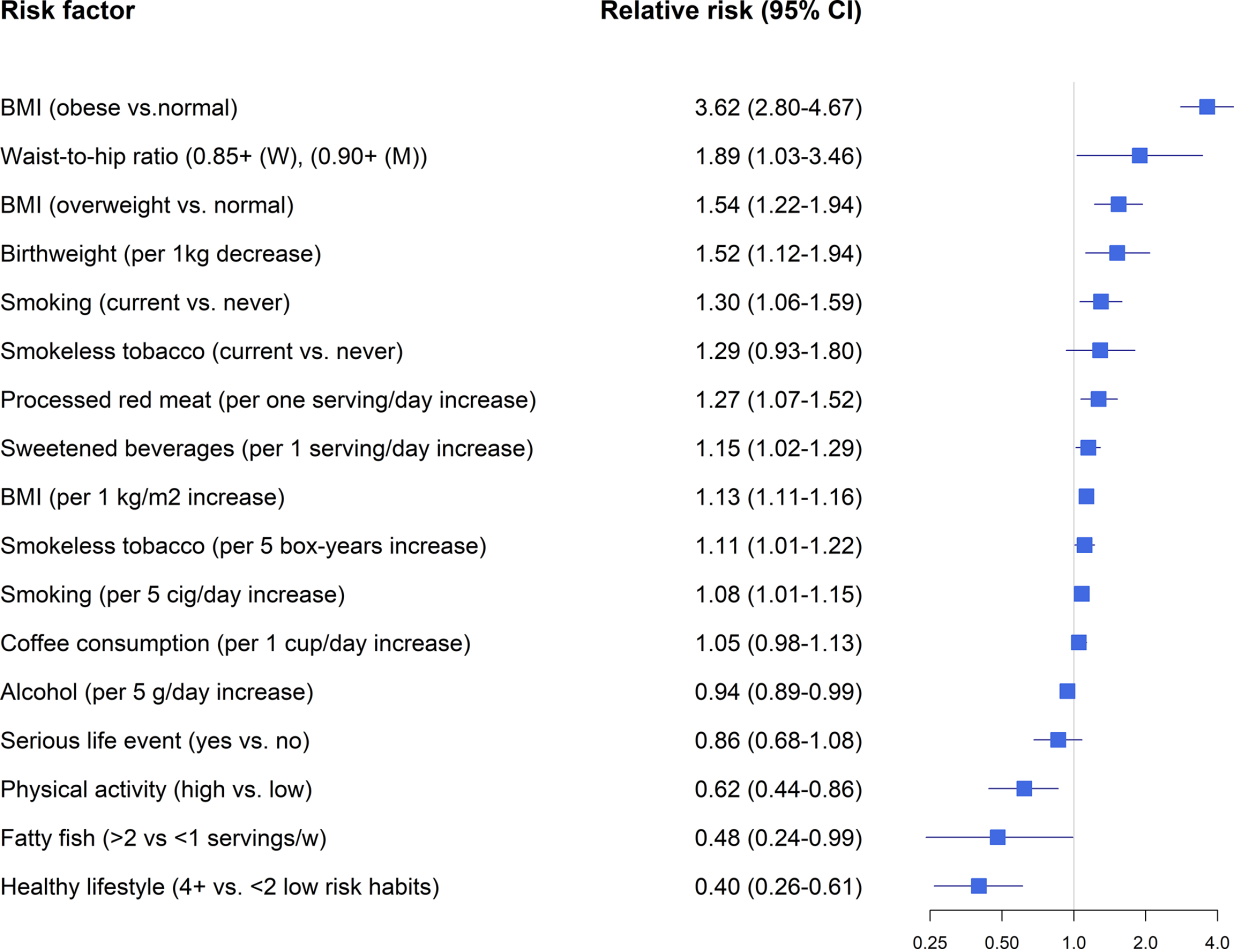
Lifestyle factors and LADA-findings from observational studies. Relative risk estimates extracted from references (28, 36, 39–47).

**Figure 2:**
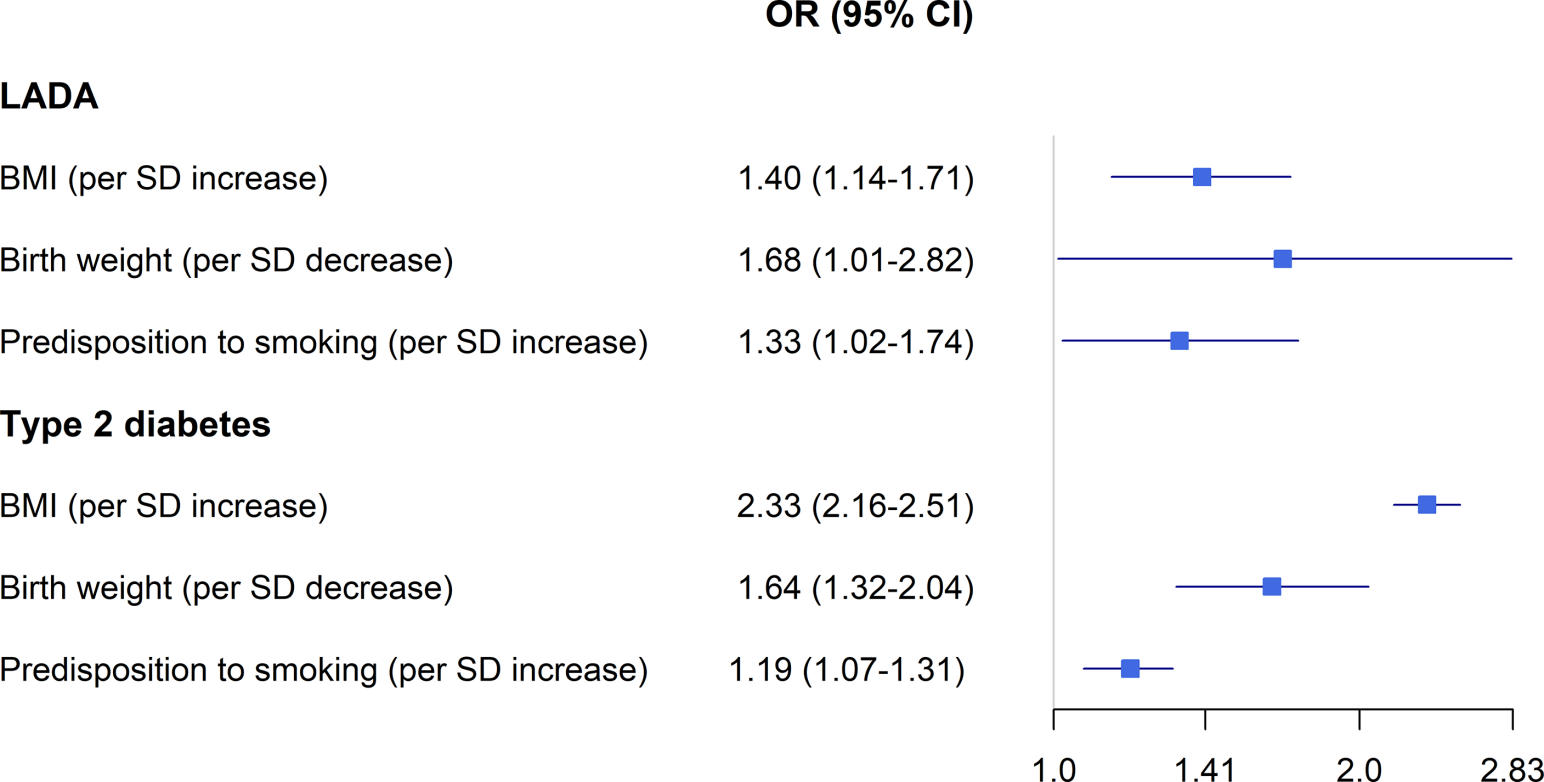
BMI, birth weight, smoking and the risk LADA compared to type 2 diabetes-findings from Mendelian randomization studies. Odds ratios extracted from references (36) and (48).

Findings from the Swedish ESTRID and Norwegian HUNT-studies will form the backbone of this review (results summarized in **Figure 1**) together with results from two recently published mendelian randomization studies (results summarized in **Figure 2**) (36, 48). In some papers, study populations overlapped. In such instances, results from the most recent publication are presented, and if there were separate effect estimates for the same exposure in different publications, those were pooled to get an overall estimate of the associations. To address the heterogeneity of LADA, some studies reported results separately for LADA with high vs. low GADA levels, divided by the median (36, 39–42, 49). These subgroups will be referred to as LADA^high^ and LADA^low^. A handful of studies included data on interaction with genetic risk factors, primarily HLA risk genotypes (28, 36, 40, 43, 48, 50, 51). In these studies, carriers of HLA high risk had genotypes DR3/3, DR3/4, DR4/4, or haplotypes of DR4-DQ8 or DR3-DQ2 and carriers of HLA low/intermediate risk had DR3/X, DR4/X, DRX/X or DR4-DQ7.

### BMI and Physical Activity

Overweight and obesity are the major modifiable risk factors for type 2 diabetes (52). Pooled data from the ESTRID and HUNT-studies indicates that obesity is associated with a 3.6-fold increased risk of LADA and every additional BMI unit or WHR unit confers a RR of 1.54 and 1.89, respectively (**Figure 1**) (49). Recently, we confirmed the positive association between BMI and LADA risk using Mendelian Randomization design (48) (**Figure 2**). This is in line with cross-sectional studies that consistently show that LADA patients have a higher BMI than those with type 1 diabetes but a lower BMI than those with type 2 diabetes (19, 32, 53). In Epic-InterAct, a large European multicenter study, Rolandsson et al. discovered higher mean BMI levels in GADA positive individuals who progressed to diabetes compared to those that did not (28.4 vs. 25.8) (54). A Chinese study did not observe any difference in baseline BMI between GADA positive individuals who developed diabetes during a five-year follow-up period vs. those who did not (55). This observation was, however, based on small numbers (13 GADA positive individuals with diabetes) and did not account for potential confounders such as age and sex. Overweight is a main cause of insulin resistance, and in line with this, we observe that BMI is positively associated with HOMA-IR in LADA (49). Adiposity may also play a role in the pathogenesis of LADA by promoting inflammation and beta-cell stress. The relative importance of BMI in the promotion of diabetes seems to vary by degree of autoimmunity, so that the association is stronger for type 2 diabetes than for LADA and stronger for LADA with low compared to high GADA levels (49). Another possibility is that high BMI promotes autoimmunity and adds to the genetically induced autoimmune reactivity. Interestingly, childhood adiposity has been implicated in the promotion of type 1 diabetes according to both observational studies (56) and a Mendelian randomization study (57). This indicates that overweight/obesity plays a role in the promotion of all three forms of diabetes and suggests that major public health gains could be made through the prevention of overweight. Based on ESTRID and HUNT data, 31-56% of all LADA and 70-82% of all type 2 diabetes were estimated to be attributable to overweight (49).

Physical activity stimulates insulin sensitivity and may prevent obesity and subsequent type 2 diabetes (58). Physical activity is also associated with a reduced risk of LADA in ESTRID and HUNT-studies (50). The association was attenuated substantially once BMI was accounted for, which could imply that any beneficial effects on LADA are mediated through reductions in adiposity (50). It has not been investigated if physical activity may also reduce the risk of type 1 diabetes (11).

### Low Birth Weight

Data from the ESTRID-study, based on a relatively small number of cases (134 LADA cases collected 2010-2014), reveals an increased risk of LADA in relation to low birth weight (44), in line with observations in type 2 diabetes (59). We could recently confirm this association using a Mendelian Randomization design (48) (**Figure 2**). The mechanism linking low birth weight to an excess risk of type 2 diabetes is not clear, but according to the “thrifty phenotype hypothesis” (60), poor fetal nutrition may lead to metabolic disturbances and a phenotype adapted to saving energy that is prone to obesity and type 2 diabetes. In support of this hypothesis, we find that the combination of low birth weight and adult overweight confers a particularly high risk of both LADA and type 2 diabetes (44). The results of our MR study in LADA (48) and previous MR (61) and twin (62) studies in type 2 diabetes indicate that link between low birth weight and diabetes is due to environmental rather than genetic factors. The risk of type 1 diabetes is slightly increased in individuals with a high rather than low birth weight, which may reflect effects of maternal glycemia on fetal growth (63).

### Tobacco

Smoking is associated with an increased risk of type 2 diabetes (64). In contrast, a reduced risk of type 1 diabetes is consistently observed in the offspring of mothers who smoked during pregnancy (65). Earlier findings based on the Norwegian HUNT-study and data collected between 1984 and 2008 suggested that smokers may have a reduced risk of LADA (66). However, updated analyses based on pooled data from ESTRID and HUNT studies, with longer follow-up time and eight times as many LADA cases, indicate an increased risk of LADA in smokers that increases with the number of cigarettes consumed per day (36). The possible adverse effects of smoking on the development of LADA are supported by findings from our MR study (36). There is also an increased risk of LADA in users of a Swedish smokeless tobacco (36). This tobacco product is commonly used in Scandinavia and associated with an elevated risk of type 2 diabetes (67). Swedish smokeless tobacco has similar nicotine content as cigarettes, but much lower levels of other potentially harmful chemicals found in tobacco smoke, such as nitrosamines (68). Given that nicotine has been shown to reduce insulin sensitivity (69), this supports the hypothesis that tobacco use, by way of increasing insulin resistance, contributes to the development of LADA. In support of this, there was a positive association between pack-years of smoking and insulin resistance (HOMA) in LADA patients from the ESTRID and HUNT studies (36).

### Diet

There is some indication that diet may influence the risk of LADA, beyond any effects mediated through effects on BMI; Findings based on the ESTRID-study indicate an elevated risk of LADA in relation to high intake of sweetened beverages (45) and processed red meat (43), whereas a reduced risk is noted for high consumption of fatty fish (46). A shortage of data has made it difficult to confirm these findings in additional cohorts (the HUNT-study has limited information on diet, especially before 2006) and these findings should clearly be interpreted with caution. Still, support for a beneficial effect of fatty fish intake is provided by the European Inter-Act study, where GADA positive individuals with high plasma levels of omega-3 fatty acids that are abundant in fatty fish or high self-reported fatty fish intake, had a lower risk of progressing to diabetes than those with low levels (70). A link between fatty fish/omega-3 fatty acids and LADA seems biologically plausible since omega-3 fatty acids, particularly those of marine origin, have anti-inflammatory and immunomodulatory properties (71). Some studies have also linked omega-3 fatty acids to a reduced risk of type 1 diabetes, although the findings have not been conclusive (72). Intake of fish does not seem to influence the risk of type 2 diabetes according to observational studies (73) and an MR study that did not find evidence of a protective effect of long-chain omega-3 fatty acids on type 2 diabetes risk (74). Intake of sweetened beverages is associated with an increased risk of type 2 diabetes (75), and total sugar intake has also been linked to the risk of type 1 diabetes (76). The mechanism may involve the promotion of overweight and direct adverse effects on glucose metabolism and insulin sensitivity (77). Findings regarding processed red meat and elevated LADA risk also coincide with observations in type 2 diabetes (73). Some studies have also linked intake of meat and nitrites, which are abundant in processed red meat, to the risk of type 1 diabetes (72). Processed red meat may promote diabetes by promoting insulin resistance and through detrimental effects on the beta-cells (78).

### Coffee and Alcohol

Moderate alcohol consumption is associated with a reduced risk of type 2 diabetes and a similar association was observed with LADA both in the HUNT (47) and ESTRID-studies (42). According to pooled data from these studies, every 5 grams of alcohol consumed per day reduced the risk of LADA by 6%. These findings may reflect beneficial effects of ethanol on insulin sensitivity (79) and inflammation (80). It should be noted, however, that the inverse association was only seen for LADA^low^ and there was nothing to suggest that alcohol would prevent autoimmunity or reduce the risk of LADA^high^, neither in the Swedish nor Norwegian data (42, 47). One interpretation of this finding is that any beneficial effects of alcohol intake on insulin sensitivity will not be enough to prevent progression to diabetes in individuals with more pronounced autoimmune -induced insulin deficiency. In contrast, Swedish data indicated that coffee consumption was associated with an increased risk of LADA^high^ but not with LADA^low^ (40). There was also an inverse association between the number of cups of coffee consumed per day and beta-cell function (HOMA). This conflicts with findings in type 2 diabetes (81) but is in accordance with a previous study in type 1 diabetes; Virtanen et al. observed an increased risk in adolescents who consumed ≥2 cups of coffee per day (82). Some components of coffee may have immunomodulatory effects or increase proinflammatory markers that promote autoimmunity (83). This may be a chance finding, but the potential adverse effects of coffee consumption on autoimmunity warrant further investigation.

### Healthy Lifestyle

Unhealthy lifestyle factors play a key role in the promotion of type 2 diabetes; a meta-analysis based on 14 observational studies found a 75% reduced risk in individuals with the healthiest compared to the unhealthiest lifestyle (using information on BMI, smoking, alcohol, physical activity, and diet) (84). Randomized controlled trials confirm the beneficial effect of lifestyle modification on type 2 diabetes risk (52) while it is unknown how to prevent type 1 diabetes (11). Regarding LADA, Swedish data indicate that the risk can be reduced by 60% through an overall healthy lifestyle including normal weight, non-smoking, physical activity, moderate alcohol consumption, and a healthy diet (39). Among the individual items, the largest risk reduction comes from maintaining a healthy weight. Importantly, a healthy lifestyle was also associated with a reduced risk of LADA in individuals with high-risk HLA genotypes or family history of diabetes. In addition, the risk reduction applied to more autoimmune LADA; a 50% reduced risk of LADA^high^ was observed in individuals with a healthy compared to unhealthy lifestyle (39).

## Interaction Between Lifestyle and Genetic Susceptibility

**Figure 3:**
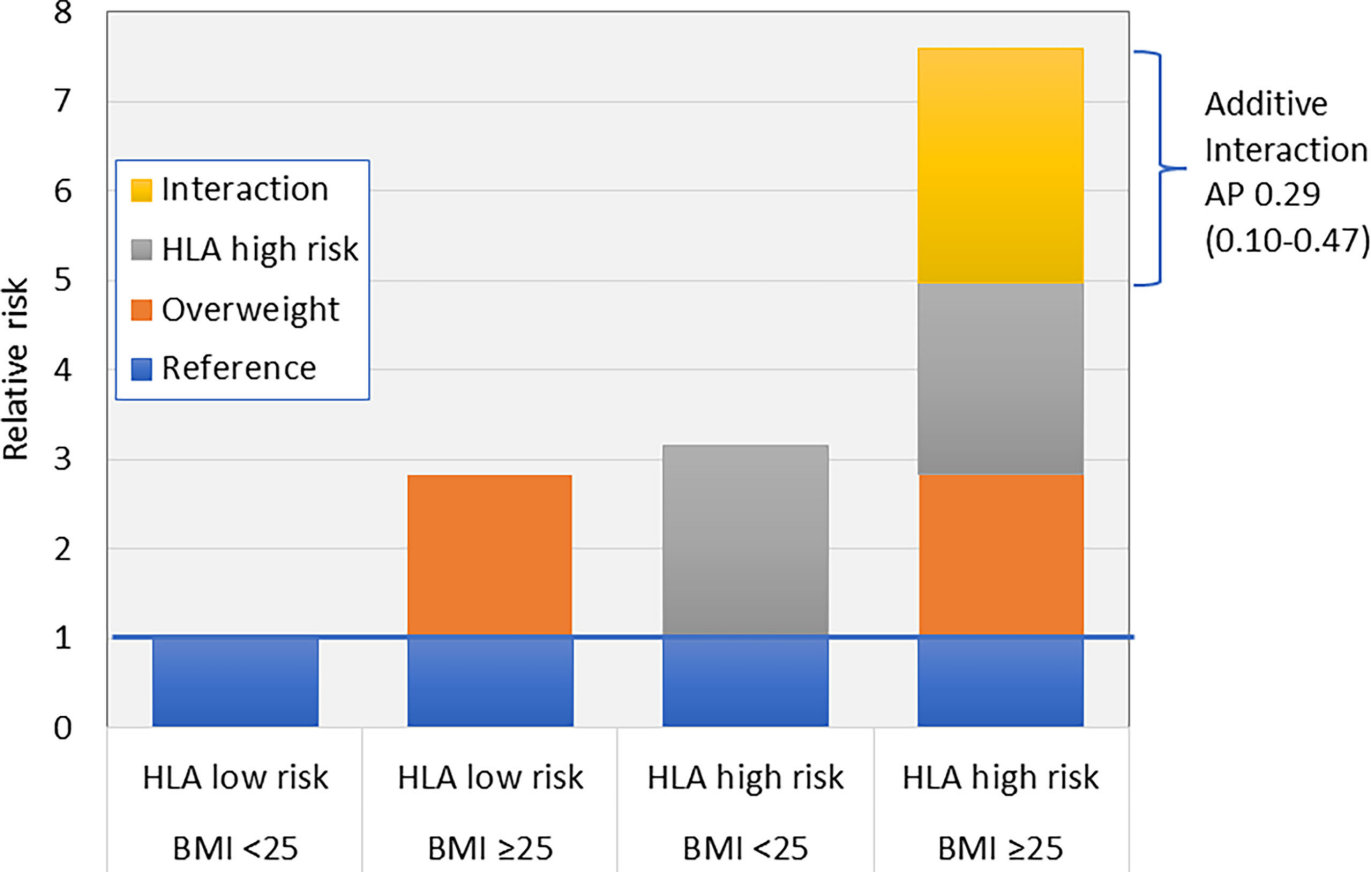
Additive interaction between HLA risk genotypes and overweight (BMI >25) on the risk of LADA (28). AP, Attributable proportion due to interaction.

A few studies have addressed the presence of interaction between lifestyle factors and genetic susceptibility in LADA, focusing primarily on carriers of HLA high-risk genotypes (an overview of these studies is presented in **Table 1**). These studies have used departure from additivity of effects, also known as additive interaction, as a criterion (85). Additive interaction is present if the risk of developing the disease, e.g., LADA, is greater in individuals exposed to both risk factors, i.e., genetic susceptibility and lifestyle exposure, than the sum of the effects of each individual factor. The magnitude of additive interaction can be assessed by the attributable proportion due to interaction (AP), which is an estimate of the proportion of cases that can be attributed to the combined effect of both risk factors. The principle of additive interaction is demonstrated in **Figure 3** using data on the combination of overweight and HLA risk genotypes in relation to LADA (28) as an example.

**Table 1:** Summary of tentative lifestyle risk factors for LADA and their potential interaction with HLA risk genes.

	Level ofcertainty for overall association*	Overall Relative risk(95% CI)	Interaction with HLAgenotype	CombinedRelative risk (95% CI)	Attributable proportion due to interaction(95% CI)	Reference
**Smoking** (current vs. never)	+++	1.30 (1.06-1.59)	Yes	3.6 (2.59−5.00)	0.27 (0.01–0.53)	Edstorp et al. (36)
**Smokeless tobacco** (current vs. never)	+	1.29 (0.93–1.80)	Possibly	6.65 (3.31–13.36)	0.42 (-0.01–0.85)	Edstorp et al. (36)
**Overweight** (BMI ≥25 vs. <25)	+++	2.26 (1.90-2.68)	Yes	7.59 (5.27–10.93)	0.29 (0.10-0.47)	Hjort et al. (28)
**Processed red meat** (>0.3 vs. ≤0.3 servings/day)	+	1.37 (1.13-1.66)	yes	8.05 (4.86-13.4)	0.53 (0.32 -0.73)	Löfvenborg et al. (43)
**Coffee** (≥ 4 vs. < 2 cups/day)	+	1.21 (0.90-1.62)	Yes	5.74 (3.34–9.88)	0.36 (0.01–0.71)	Rasouli et al. (40)
**Sweetened beverages** (>2 vs. 0 servings/day)	+	2.04 (1.11-3.77)	No			Löfvenborg et al. (51)
**Physical activity** (high vs. low)	++	0.61 (0.43-0.86)	No	–	–	Hjort et al. (50)
**Low birth weight** (<3 kg vs. ≥4 kg)	+	2.38 (1.23-4.60)	?	–	–	Hjort et al. (44)
**Alcohol** (per 5 g increase/day)	++	0.94 (0.89–0.99)	?	–	–	Rasouli et al. (47), Rasouli et al. (42)
**Fatty fish** (>2 vs <1 servings/week)	++	0.48 (0.24–0.99)	?	–	–	Löfvenborg et al. (46)

*Level of certainty for overall association: +++ observational findings supported by MR results, ++ supported by two observational studies, + only one observational study. AP-attributable proportion due to interaction.

Studies conducted to date indicate that overweight, smoking, high intake of coffee, and processed red meat, interact with HLA DR-DQ genotypes in LADA, conferring much higher risks among those exposed to both factors (**Table 1**). Nothing suggests that the influence of high intake of sugar sweetened beverages or low physical activity on LADA risk is magnified in carriers of HLA-risk genotypes. On the contrary, these associations were primarily observed in non-carriers of these genotypes (50, 51). Whether alcohol consumption, low birth weight, and fatty fish intake interact with genetic susceptibility in LADA has not been investigated.

There is some indication that the influence of lifestyle factors varies across specific HLA-risk genotypes, e.g., overweight individuals with any HLA-risk genotype have 7.8 times the risk of LADA compared to normal weight individuals with low or intermediate genotypes, whereas being homozygous for the DR4 genotype and overweight confers an odds ratio of 26.8 (95% CI 15.4-46.4) with 58% (95% CI 0.32-0.83) of the excess risk attributable to additive interaction (28). Interaction between other lifestyle factors and specific HLA genotypes has not been addressed in LADA but is an important area for future studies since this may allow us to identify the individuals who would benefit the most from lifestyle modification, e.g., weight reduction or smoking cessation.

We are not aware of any study addressing additive interactions between HLA genotypes and environmental exposures in relation to type 1 diabetes. However, the excess risk of type 1 diabetes related to intake of red meat (86) and sweetened beverages (76) was observed in children with high genetic risk. There is also some evidence that HLA genotypes may modify the influence of obesity on type 1 diabetes risk in children; TrialNet findings show that BMI increases the rate of progression to multiple antibodies primarily in children who do not carry HLA risk genotypes (87). Similarly, a Swedish case-only study found a higher prevalence of obesity in children with type 1 diabetes and low-risk HLA genotypes (88). Unfortunately, the numbers provided (87) and the lack of controls (88) preclude calculation of the combined effect of HLA risk genotypes and obesity on type 1 diabetes risk.

The potential interaction between risk variants of *TCF7L2* and the fat mass and obesity-associated (*FTO*) gene and overweight (28), sugar sweetened beverages (51), red meat (43) and physical activity (50) on the risk of LADA has also been investigated. Additive interaction with overweight was observed for *TCF7L* (combined RR=2.65, 95% CI 1.97-3.65, AP=0.31, 95% CI 0.09-0.52) and *FTO* (combined RR=2.21, 95% CI 1.60-3.07, AP=0.38, 95% CI 0.15-0.61) variants, but not for the other lifestyle exposures.

## Etiology of LADA

**Figure 4:**
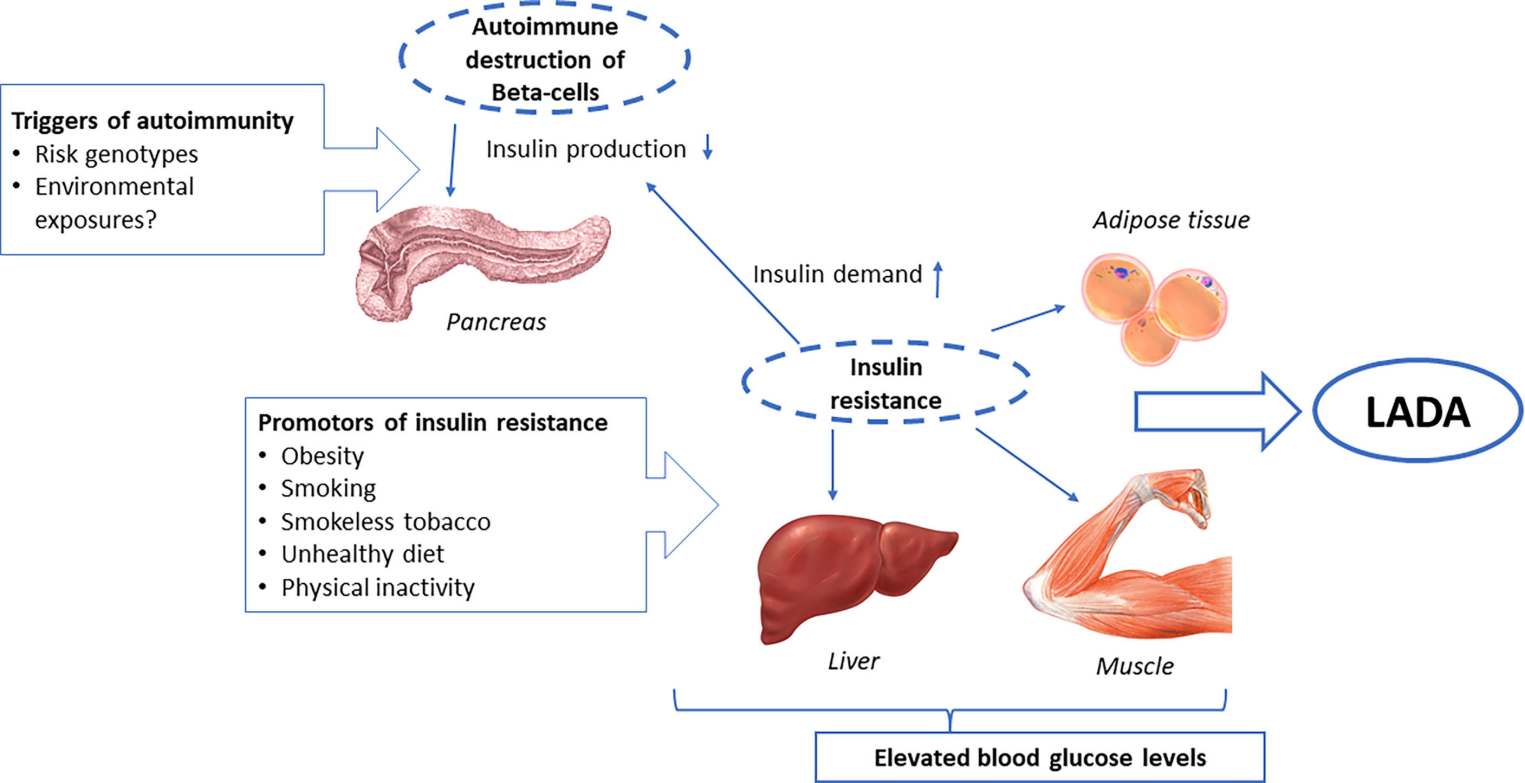
Etiology of LADA.

Studies on genetic and lifestyle influences on LADA confirm its hybrid nature, with an etiology that encompasses risk factors for both type 1 diabetes and type 2 diabetes. On one hand, there are clear genetic similarities between LADA and type 1 diabetes, with a high risk conferred primarily by HLA DR-DQ genotypes (6). This stresses the autoimmune nature of LADA, which is manifested by the presence of autoantibodies and insulin deficiency. On the other hand, lifestyle risk factors for LADA seem to primarily include factors also linked to type 2 diabetes and insulin resistance, such as overweight/obesity, smoking, and low birth weight. These findings suggest that the pathogenesis of LADA includes autoimmune destruction of the beta-cells that at least partly has a genetic origin, together with insulin resistance, induced by lifestyle factors (**Figure 4**). Acquired insulin resistance in skeletal muscles, liver, and adipose tissue may contribute to the promotion of LADA by increasing the demand on the beta-cells to increase insulin production. If such a compensatory rise is not possible due to autoimmune destruction of the beta-cells, glucose levels will rise and, eventually, LADA will become manifest (**Figure 4**). This fits with the accelerator hypothesis, which proposes that obesity-induced insulin resistance plays a key role in the development of type 1 diabetes by accelerating progression to hyperglycemia in genetically susceptible individuals (89). There is some evidence of interaction between lifestyle factors, such as smoking and obesity, and HLA DR-DQ genotypes in the promotion of LADA. This could reflect that the adverse effect of acquired insulin resistance is greater in individuals with genetic susceptibility to autoimmunity. It is also possible that, e.g., obesity or smoking, directly affects the immune system and intensifies the autoimmune response in individuals with genetically caused autoimmunity.

Whether environmental factors may also trigger autoimmunity is not clear. The considerable rise in the incidence of type 1 diabetes in children observed in children over the last 30 years must be due to environmental changes and suggests that autoimmunity may have environmental origins. Such risk factors have proved to be elusive. The environmental factors most consistently linked to type 1 diabetes-risk are virus infections, psychosocial stress, and some dietary exposures (11, 90). With regard to psychosocial factors, we did not find an increased risk of LADA in individuals exposed to serious life events such as death in the family, divorce, or serious illness (41), and exposure to viral infections has so far not been investigated in relation to LADA. There is some indication that dietary factors may influence the risk of LADA. Interestingly, there are findings proposing beneficial effects of fatty fish intake and adverse effects of coffee consumption on the development of autoimmunity, although it is still too early to draw any firm conclusions as to whether such factors may modify the risk of LADA.

## Future Direction

The evidence linking lifestyle factors to LADA risk is generally weak as it is mostly based on only one or two observational studies conducted in Scandinavian populations. Confirmations are clearly warranted, and importantly, extensions to other ethnic groups. There is also a need for larger studies, since those conducted to date are hampered by small numbers, especially when addressing gene*environment interactions. With larger studies, we can also address interactions between sets of environmental risk factors. Importantly, two-sample MR-studies support a causal effect of adiposity, low birth weight, and smoking on the development of LADA. Notably, these studies used GWAS data from populations of European ancestry, and whether they can be generalized to other ethnic groups is not clear. Further MR-studies in LADA are an important complement to observational studies in order to make stronger causal inferences. At present, the possibility to perform MR studies is limited since there is currently only one GWAS in LADA available (27). There are several potential lifestyle or environmental risk factors that remain to be investigated in LADA, including virus infections as well as further investigations into the role of diet, both for confirmation and for exploration of additional dietary factors linked to either type 1 diabetes or type 2 diabetes, such as intake of dairy products, whole grains, fruit, and vegetables, as well as dietary patterns like the Mediterranean diet. These investigations should ideally be based on objectively measured dietary exposures and careful consideration of potential confounders. Studies on gene*environment in LADA conducted so far have primarily focused on HLA genotypes and dichotomized categorizations into high and low genetic risk. To further our understanding of these proposed interactions, investigations into the role of specific HLA risk genotypes are needed, and for this to be feasible, we need larger data sets. The interaction between lifestyle and susceptibility to type 2 diabetes in LADA also needs to be addressed. Such studies may benefit from using polygenetic risk scores, which will be better at capturing susceptibility to type 2 diabetes than individual risk variants. In this context, it is also noteworthy that there is a paucity of studies on environmental risk factors for adult-onset type 1 diabetes. The results of such studies would be valuable to compare with the findings in LADA. At present, only a few, mostly small prospective studies (91–94) have addressed the long-term prognosis of LADA and the role of different prognostic factors is largely unknown. There is a need for investigations into the role of treatment and lifestyle for the risk of complications in LADA. Such knowledge may lead to improved secondary prevention.

## Conclusion

Studies conducted to date indicate that unhealthy lifestyle factors, including overweight, smoking, low physical activity, and high intake of processed red meat and sweetened beverages, contribute to the development of LADA and, furthermore, that the adverse effects of some of these factors, especially smoking and being overweight, may be more pronounced in individuals with genetic susceptibility to autoimmunity. The preventive potential of LADA appears to be substantial, and the largest health gains will most likely be made through the prevention of overweight. Overweight and obesity are major causes of type 2 diabetes and are also linked to type 1 diabetes. This implies that the incidence of all three major forms of diabetes would be reduced if we managed to successfully target obesity through public health interventions. Informing individuals with a family history of diabetes regarding the impact of being overweight on their risk of diabetes is also essential. Research on the role of lifestyle factors in the development of LADA is however in its infancy and future investigations in this field are urgently needed.

## Author Contributions

SC conceived and wrote the manuscript and agreed to be accountable for its content.

## Funding

SC was supported by grants from the Swedish Research Council (2018-03035), the Research Council for Health, Working Life and Welfare (FORTE, 2018-00337), and the Novo Nordisk Foundation (NNF19OC0057274).

## Conflict of Interest

The author declares that the research was conducted in the absence of any commercial or financial relationships that could be construed as a potential conflict of interest.

## Publisher’s Note

All claims expressed in this article are solely those of the authors and do not necessarily represent those of their affiliated organizations, or those of the publisher, the editors and the reviewers. Any product that may be evaluated in this article, or claim that may be made by its manufacturer, is not guaranteed or endorsed by the publisher.
