# Supplementary material for: Lifestyle or Environmental Influences and Their Interaction With Genetic Susceptibility on the Risk of LADA
Source: Front Endocrinol (Lausanne). 2022 Jun 29;13:917850. doi: 10.3389/fendo.2022.917850 (PMC9276967; doi:10.3389/fendo.2022.917850)
Supplement: Supplementary file 1 [file Table_1.docx]

### Appendix to Carlsson 2022.

### Studies investigating the risk of LADA in relation to lifestyle factors. Results of PubMed search Dec 2021.

1. Herzog K, Ahlqvist E, Alfredsson L, Groop L, Hjort R, Löfvenborg JE, Tuomi T, Carlsson S. Combined lifestyle factors and the risk of LADA and type 2 diabetes - Results from a Swedish population-based case-control study. Diabetes Res Clin Pract. 2021 Apr;174:108760.
2. Hjort R, Ahlqvist E, Andersson T, Alfredsson L, Carlsson PO, Grill V, Groop L, Martinell M, Sørgjerd EP, Tuomi T, Åsvold BO, Carlsson S. Physical Activity, Genetic Susceptibility, and the Risk of Latent Autoimmune Diabetes in Adults and Type 2 Diabetes. J Clin Endocrinol Metab. 2020 Nov 1;105(11):e4112–23.
3. Löfvenborg JE, Ahlqvist E, Alfredsson L, Andersson T, Groop L, Tuomi T, Wolk A, Carlsson S. Consumption of red meat, genetic susceptibility, and risk of LADA and type 2 diabetes. Eur J Nutr. 2021 Mar;60(2):769-779.
4. Hjort R, Löfvenborg JE, Ahlqvist E, Alfredsson L, Andersson T, Grill V, Groop L, Sørgjerd EP, Tuomi T, Åsvold BO, Carlsson S. Interaction Between Overweight and Genotypes of HLA, TCF7L2, and FTO in Relation to the Risk of Latent Autoimmune Diabetes in Adults and Type 2 Diabetes. J Clin Endocrinol Metab. 2019 Oct 1;104(10):4815-4826.
5. Löfvenborg JE, Ahlqvist E, Alfredsson L, Andersson T, Dorkhan M, Groop L, Tuomi T, Wolk A, Carlsson S. Genotypes of HLA, TCF7L2, and FTO as potential modifiers of the association between sweetened beverage consumption and risk of LADA and type 2 diabetes. Eur J Nutr.2020 Feb;59(1):127-135.
6. Rasouli B, Ahlqvist E, Alfredsson L, Andersson T, Carlsson PO, Groop L, Löfvenborg JE, Martinell M, Rosengren A, Tuomi T, Wolk A, Carlsson S. Coffee consumption, genetic susceptibility and risk of latent autoimmune diabetes in adults: A population-based case-control study. Diabetes Metab. 2018 Sep;44(4):354-360. doi: 10.1016/j.diabet.2018.05.002. Epub 2018 May 17. PubMed PMID: 29861145.
7. Hjort R, Ahlqvist E, Carlsson PO, Grill V, Groop L, Martinell M, Rasouli B, Rosengren A, Tuomi T, Åsvold BO, Carlsson S. Overweight, obesity and the risk of LADA: results from a Swedish case-control study and the Norwegian HUNT Study. Diabetologia. 2018 Jun;61(6):1333-1343. doi: 10.1007/s00125-018-4596-0. Epub 2018 Mar 27. PubMed PMID: 29589073; PubMed Central PMCID: PMC6448998.
8. Rasouli B, Andersson T, Carlsson PO, Hjort R, Löfvenborg JE, Martinell M, Groop L, Tuomi T, Carlsson S. Serious life events and the risk of latent autoimmune diabetes in adults (LADA) and Type 2 diabetes. Diabet Med. 2017;34:1259-1263.
9. Rasouli B, Andersson T, Carlsson PO, Grill V, Groop L, Martinell M, Midthjell K, Storm P, Tuomi T, Carlsson S. Use of Swedish smokeless tobacco (snus) and the risk of Type 2 diabetes and latent autoimmune diabetes of adulthood (LADA). Diabet Med. 2017;34:514-521.
10. Rasouli B, Andersson T, Carlsson PO, Grill V, Groop L, Martinell M, Storm P, Tuomi T, Carlsson S. Smoking and the Risk of LADA: Results From a Swedish Population-Based Case-Control Study. Diabetes Care. 2016;39:794-800.
11. Löfvenborg JE, Andersson T, Carlsson PO, Dorkhan M, Groop L, Martinell M, Tuomi T, Wolk A, Carlsson S. Sweetened beverage intake and risk of latent autoimmune diabetes in adults (LADA) and type 2 diabetes. Eur J Endocrinol. 2016;175:605-614.
12. Hjort R, Alfredsson L, Carlsson PO, Groop L, Martinell M, Storm P, Tuomi T and Carlsson S. Low birth weight is associated with an increased risk of Latent Autoimmune Diabetes in Adults (LADA) and type 2 diabetes: Results from a Swedish case-control study. Diabetologia. 2015 Nov;58(11):2525-32. doi: 10.1007/s00125-015-3711-8. Epub 2015 Jul 25.
13. Löfvenborg JE, Andersson T, Carlsson PO, Dorkhan M, Groop L, Martinell M, Tuomi T, Wolk A, Carlsson S. Fatty fish consumption and risk of latent autoimmune diabetes in adults. Nutr Diabetes. 2014 Oct 20;4:e139. doi: 10.1038/nutd.2014.36.
14. Rasouli B, Andersson T, Carlsson PO, Dorkhan M, Grill V, Groop L, Martinell M, Tuomi T, Carlsson S. Alcohol and the risk for latent autoimmune diabetes in adults: results based on Swedish ESTRID study. Eur J Endocrinol. 2014;171:535-43.
15. Löfvenborg JE, Andersson T, Carlsson PO, Dorkhan M, Groop L, Martinell M, Rasouli B, Storm P, Tuomi T, Carlsson S. Coffee consumption and the risk of latent autoimmune diabetes in adults--results from a Swedish case-control study. Diabet Med. 2014;31:799-805.
16. Rasouli B, Grill V, Midthjell K, Ahlbom A, Andersson T, Carlsson S. Smoking is associated with reduced risk of autoimmune diabetes in adults contrasting with increased risk in overweight men with type 2 diabetes: a 22-year follow-up of the HUNT study. Diabetes Care. 2013 Mar;36(3):604-10.
17. Rasouli B, Ahlbom A, Andersson T, Grill V, Midthjell K, Olsson L, Carlsson S. Alcohol consumption is associated with reduced risk of Type 2 diabetes and autoimmune diabetes in adults: results from the Nord-Trøndelag health study. Diabet Med. 2013 Jan;30(1):56-64.
18. Carlsson S, Midthjell K, Tesfamarian MY, Grill V. Age, overweight and physical inactivity increase the risk of latent autoimmune diabetes in adults: results from the Nord-Trøndelag health study. Diabetologia. 2007;50:55-8.
19. Carlsson S, Midthjell K, Grill V; Nord-Trøndelag study. Smoking is associated with an increased risk of type 2 diabetes but a decreased risk of autoimmune diabetes in adults: an 11-year follow-up of incidence of diabetes in the Nord-Trøndelag study. Diabetologia. 2004;47:1953-6.
